# Autophagy in Rare (NonLysosomal) Neurodegenerative Diseases

**DOI:** 10.1016/j.jmb.2020.02.012

**Published:** 2020-04-03

**Authors:** Malgorzata Zatyka, Sovan Sarkar, Timothy Barrett

**Affiliations:** 1 Institute of Cancer and Genomic Sciences, College of Medical and Dental Sciences, University of Birmingham, Birmingham, B15 2TT, UK; 2 Department of Endocrinology, Birmingham Women's and Children's Hospital, Steelhouse Lane, Birmingham B4 6NH, UK

**Keywords:** autophagy, neurodegeneration, child

## Abstract

Neurodegenerative diseases (NDDs) comprise conditions with impaired neuronal function and loss and may be associated with a build-up of aggregated proteins with altered physicochemical properties (misfolded proteins). There are many disorders, and causes include gene mutations, infections, or exposure to toxins. The autophagy pathway is involved in the removal of unwanted proteins and organelles through lysosomes. While lysosomal storage disorders have been described for many years, it is now recognised that perturbations of the autophagy pathway itself can also lead to neurodegenerative disease. These include monogenic disorders of key proteins involved in the autophagy pathway, and disorders within pathways that critically control autophagy through monitoring of the supply of nutrients (mTORC1 pathway) or of energy supply in cells (AMPK pathway). This review focuses on childhood-onset neurodegenerative disorders with perturbed autophagy, due to defects in the autophagy pathway, or in upstream signalling via mTORC1 and AMPK. The review first provides a short description of autophagy, as related to neurons. It then examines the extended role of autophagy in neuronal function, plasticity, and memory. There follows a description of each step of the autophagy pathway in greater detail, illustrated with examples of diseases grouped by the stage of their perturbation of the pathway. Each disease is accompanied by a short clinical description, to illustrate the diversity but also the overlap of symptoms caused by perturbation of key proteins necessary for the proper functioning of autophagy. Finally, there is a consideration of current challenges that need addressing for future therapeutic advances.

## Highlights

• Autophagy is an important pathway for the removal of misfolded proteins from terminally differentiated neurons.
• Monogenic defects in autophagy cause childhood-onset neurodegeneration.
• Defects in different stages of the pathway may present with overlapping clinical features.
• Increasing autophagic flux may be a therapeutic strategy to treat many autophagic disorders.

## Graphical abstract

**Figure:**
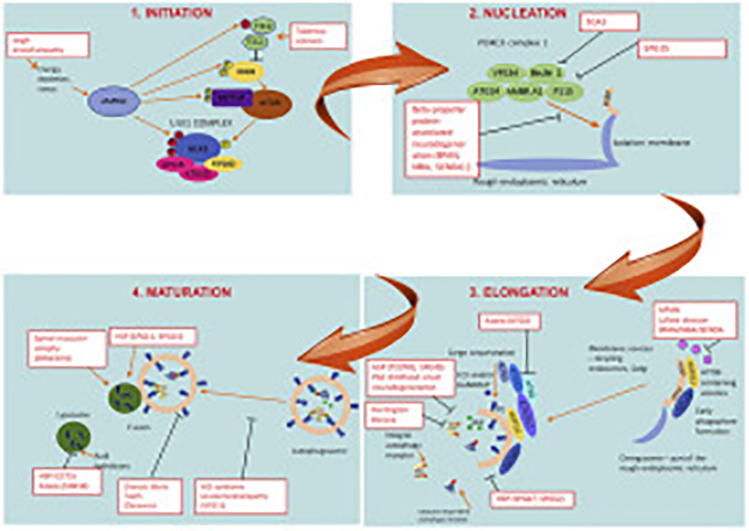

## Introduction

Neurodegenerative diseases (NDDs) comprise conditions with impaired neuronal function and loss and may be associated with a build-up of aggregated proteins with altered physicochemical properties (misfolded proteins). It has been estimated that almost 30% of newly translated proteins in the cell are not folded correctly, even under healthy conditions [1]. The classic pattern of a neurodegenerative disorder in childhood is regression and progressive loss of neuronal function affecting speech, vision, hearing, or movement. It may be accompanied by seizures and cognitive decline [2]. There are many disorders that can be caused by monogenic defects, infections, or exposure to toxic substances. Anatomically, they may involve the neuronal cell bodies, both unmyelinated and myelinated axons, dendrites, and glial cells.

The realisation that many NDDs involve abnormal protein deposition in the brain led to discoveries, such as the role of the unfolded protein response [3], and protein elimination pathways, such as the ubiquitin-proteasome pathway and the autophagy pathway [4]. These pathways may interact with other pathways, such as mitochondrial energy metabolism, ion homeostasis, and intermediary metabolic pathways, to damage neurons. DNA damage, neuroinflammatory processes, and disruption of cellular/axonal transport may also result in the toxicity of NDD-related proteins [5].

The role of the autophagy pathway in neurodegenerative diseases has received prominence as a route for clearance of cytosolic, aggregate-prone proteins, and organelle degradation through lysosomes [5]. Lysosomes function as the primary digestive units within cells. Their function is to break down complex components into simpler ones. Each cell has hundreds of lysosomes that degrade substrates, such as misfolded proteins and protein aggregates. When this process does not take place or is overwhelmed, a progressive accumulation of undigested macromolecules can occur predominantly in the cytosol, and even in the lysosomes causing lysosomal storage disorders (LSDs). LSDs are a group of about 60 different diseases, which are inborn errors of metabolism, resulting in the absence or deficiency of an enzyme, leading to the inappropriate build-up and storage of material in various cells of the body.

However, it is now recognised that perturbations of the autophagy pathway itself, can also lead to a neurodegenerative disease beyond lysosomal storage disorders. These include single-gene disorders of components in the autophagy pathway and disorders in pathways that critically control autophagy through the monitoring of nutrient or amino acid supply (mTORC1 pathway) or cellular ATP levels (AMPK pathway). This review will focus on childhood-onset neurodegenerative disorders with perturbed autophagy, due to defects in the autophagy pathway, or in upstream signalling via mTORC1 and AMPK. The review will first provide a brief overview of autophagy, with particular reference to neurons. It will then examine the extended role of autophagy in neuronal function, plasticity, and memory. There follows a description of each step of the autophagy pathway in greater detail, illustrated with examples of diseases grouped by the stage of their perturbation of the pathway (Table 1). Each disease is accompanied by a clinical description, to illustrate the diversity but also the overlap of symptoms caused by perturbation of different stages of the autophagy pathway. Finally, there is a consideration of current challenges that need addressing for future therapeutic advances.

**Table 1:** List of genes, associated proteins, and childhood-onset neurodegenerative diseases associated with impaired autophagy, ordered by the likely position of each main defect in the autophagy pathway. Gene and protein names as published in UniProt www.uniprot.org.

Affected stage of the autophagy pathway	Gene	Protein	Disease
Initiation signals to the ULK1 complex: the mTOR pathway	*TSC1, TSC2*	Hamartin, Tuberin	Tuberous Sclerosis
Initiation signals to the ULK1 complex: the AMPK pathway	*e.g*. *ND5*	NADH-ubiquinone oxidoreductase chain 5	Leigh encephalopathy
Phagophore nucleation	*e.g*. *ATXN3*	Ataxin-3	Spinocerebellar ataxia type 3
Early phagophore formation	*WDR45*	WD repeat domain phosphoinositide-interacting protein 4 (WIPI4)	Beta-propeller protein-associated neurodegeneration (BPAN, NBIA, SENDA)
Early phagophore formation	*e.g. C19orf12*	Protein C19orf12	Neurodegeneration with brain iron accumulation (NBIA) (mitochondrial membrane protein-associated neurodegeneration (MPAN), hereditary spastic paraplegia (SPG43))
Phagophore membrane elongation	*ATG5*	Autophagy protein 5	Hereditary childhood ataxia
Phagophore membrane elongation	*TECPR2*	Tectonin beta-propeller repeat-containing protein 2	Hereditary spastic paraplegia (SPG49)
Phagophore membrane elongation	*AP4S1*	AP-4 complex subunit sigma-1	Hereditary spastic paraplegia (SPG47, SPG52)
Phagophore membrane elongation	*WDR45*	WD repeat domain phosphoinositide-interacting protein 4 (WIPI4)	Beta-propeller protein-associated neurodegeneration (BPAN, NBIA, SENDA)
Autophagosome formation	*EPM2A, EPM2B*	Laforin, Malin	Lafora disease
Cargo recognition and delivery to degradation	*SQSTM1/P62*	Sequestosome-1	Childhood-onset neurodegeneration
Cargo recognition and delivery to degradation	*IT15*	Huntingtin	Huntington disease
Autophagosome maturation	*VPS11*	Vacuolar sorting protein VPS11	Leucoencephalopathy
Autophagosome maturation	*SPG11, ZFYVE26*	Spatacsin, Zinc finger FYVE domain-containing protein 26 (spastizin)	Hereditary spastic paraplegia (SPG11, SPG15 (SPG15 also interacts with Beclin1)
Autophagosome/lysosome fusion	*EPG5*	Ectopic p-granules protein 5 homologue	VICI syndrome
Regulation of lysosome function	*CCT5*	Chaperonin-containing T-complex protein 1 subunit epsilon	Hereditary spastic paraplegia
Regulation of lysosome function	*SNX14*	Sorting nexin-14	Ataxia
Lysosomal/autolysosomal acidification	*DNM2*	Dynamin 2	Charcot Marie Tooth disease
Transport of autolysosome cargoes	*DYNC1H1*	Dynein cytoplasmic 1 heavy chain 1	Spinal muscular atrophy

## Overview of Autophagy

Autophagy is a cellular homeostatic catabolic pathway, which functions as a quality control mechanism to clear old proteins, unwanted protein aggregates, and damaged organelles; and to recover nutrients by the degradation of these proteins and organelles during stress conditions. A basal level of autophagy is always present in all cells, but for fulfiling its role in nutrient recovery, it is induced under stress conditions, such as starvation or energy depletion. There are three forms of autophagy with different mechanisms of cargo delivery, chaperone-mediated autophagy (CMA), microautophagy, and macroautophagy [6,7]. Chaperone-mediated autophagy selectively recognises proteins with a KFERQ-like amino acid motif and uses the chaperone proteins and Lysosome Associated Membrane Protein (LAMP2A) as a receptor, to deliver proteins for lysosomal degradation. Microautophagy delivers cargo directly to lysosomes by inward invagination of the lysosomal membrane. Macroautophagy uses double-membrane vesicles (autophagosomes) to engulf the cargo. Autophagosomes undergo multistage maturation processes and eventually fuse with lysosomes, releasing their cargo for degradation. Although autophagy occurs in almost all cell types, there is cell type/tissue-specific regulation.

Macroautophagy is generally understood as a nonspecific degradation pathway (“bulk autophagy”) where a mixed cargo is engulfed and processed. There are also cargo-specific forms of autophagy, where a specific cargo is targeted, such as *mitophagy* (degradation of mitochondria; relevant to neurodegeneration [8]), *ER-phagy,* where the autophagosomes selectively include ER membranes [9], *pexophagy* and *ribophagy* for specific degradation of peroxisomes and ribosomes respectively [10,11], *aggrephagy* (relevant to neurodegeneration) targets protein aggregates [12], *lipophagy* for lipid droplets [13], and *xenophagy* degrades intracellular pathogens [6,14]. The selectivity is achieved by receptors with domains recognising the label on the cargo and domains recognising ATG8 family members (like LC3II) on autophagosome membranes. For cargo to be recognised by the receptors, it is modified, most commonly by ubiquitination. Ubiquitinated cargo is recognised by ubiquitin-binding receptors (adaptors) like p62/SQSTM1, NBR1, OPTN (optineurin), or NDP52. Other modifications are recognised by ubiquitin independent receptors like NIX, BNIP3, FUNDC1, RETREG1/FAM134B, and galectin 8 [15].

Macroautophagy (which in this review is referred to as “autophagy”) will be the main form of autophagy considered in this review as its impairment results in many different forms of neurodegeneration.

## Upstream Pathways Required to Initiate Autophagy

In cells, there exists a certain basal constitutive level of autophagy, but the process is also strictly regulated by environmental and intracellular signalling. Nutrient and energy depletion results in the activation of autophagy via two principal upstream pathways, mammalian target of rapamycin (mTORC1) [16] and AMP-activated protein kinase (AMPK) [17]. Under the fed conditions, autophagy is inhibited by active mTORC1 kinase complex; however, when nutrient deprivation is sensed, mTORC1 becomes inactive and mTORC1 sites on ULK1 (unc51-like autophagy activating kinase 1) become dephosphorylated, removing its inhibition of ULK1 complex.

Autophagy is also induced by energy depletion sensed by the AMPK pathway, which detects the ATP deficit by sensing changes in the ATP to AMP ratio. AMPK activates autophagy directly by phosphorylation of ULK1, which activates the ULK1 complex, or indirectly via inhibition of mTORC1 [18,19].

A further level of autophagy regulation takes place during autophagy and lysosomal gene transcription. Among transcription factors involved, the key role is played by TFEB (Transcription Factor EB), which regulates expression of “coordinated lysosomal expression and regulation network of genes” (CLEAR) [20,21]. The localisation and activity of TFEB are both regulated by mTOR-mediated phosphorylation: during the fed state, TFEB is negatively regulated by mTORC1 (phosphorylated) and is located in lysosomal membranes. After mTORC1 inactivation, TFEB is dephosphorylated, released from lysosomal membranes, and translocated to the nucleus where it drives transcription of target genes. A related transcription factor, MITF, performs an analogous role in melanocytes by binding the CLEAR-box elements of lysosomal and autophagosomal genes.

## Autophagosome Biogenesis and Membrane Sources for Autophagy

**Fig. 1:**
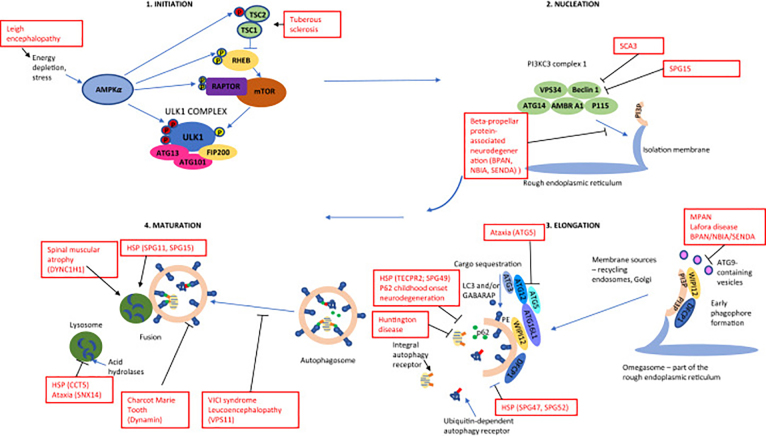
Autophagy pathway overview, with childhood-onset neurodegenerative diseases affecting the pathway either directly or indirectly highlighted in red font. Signals that activate the autophagic process (initiation) in response to stress are mediated via mTOR and AMPK pathways and colocalise at the UNC51-like kinase (ULK)1 complex. This complex consists of ULK1, autophagy-related protein 13 (ATG13), RB1-inducible coiled-coil protein 1 (FIP200), and ATG101. This complex then initiates phagophore nucleation by the phosphorylation of proteins in the PI3KC3/VPS34 complex (class III PI3 kinase PI3KC3 also known as vacuolar protein sorting 34 (VPS34), Beclin 1, ATG14, activating molecule in Beclin 1-regulated autophagy protein 1 (AMBRA1) and general vesicular transport factor (p115)). The next step is the activation of phosphatidylinositol-3-phosphate (PI3P) generation at an endoplasmic reticulum structure (Omegasome). PI3P interacts with the effector proteins WD repeat domain phosphoinositide-interacting protein (WIPI1 and 2) and zinc-finger FYVE domain-containing protein 1 (DFCP1), recruiting them to the omegasome via interaction with their PI3P-binding domains. WIPI2 interacts with the ATG12-ATG5-ATG16L1 complex. This complex facilitates ATG3-mediated conjugation of ATG8 proteins, such as microtubule-associated protein light chain 3 (LC3) proteins and gamma-aminobutyric acid receptor-associated proteins (GABARAPS) to membrane-resident phosphatidylethanoloamine (PE). This leads to the formation of lipidated forms that can bind to membranes. ATG8s attract additional proteins in the autophagy pathway containing LC3-interacting regions (LIR). They are required for membrane extension and sealing of the phagophore membrane to create the autophagosome. ATG9-containing vesicles mediate the delivery of some of these membranes. The sealing of the membrane produces the autophagosome. The next step is the removal of the ATG proteins, followed by fusion with lysosomes. Lysosomes contain acid hydrolases, which break down the autophagic cargo, allowing rescued nutrients to be returned to the cytoplasm. TSC, tuberous sclerosis complex; MPAN, mitochondrial membrane protein-associated neurodegeneration; HSP, hereditary spastic paraplegia; NBIA, neurodegeneration with brain iron accumulation. Inhibitory phosphorylation events shown as ‘P’ encircled, in yellow/light gray, activating phosphorylation events shown in red/dark gray.

Inhibition of mTORC1 results in autophagy initiation in a series of phosphorylation events of the ULK1 kinase complex proteins (ULK1, ATG13, FIP200, ATG101) leading to activation of ULK1 complex and translocation to specific sites on ER membranes called omegasomes, the platforms for phagophore formation [22]. ULK1 complex recruits the members of phosphoinositide 3-kinase (PI3K) complex (class III PI3 kinase PIK3C3/VPS34, PIK3R4/VPS15, Beclin1, ATG14, and others) to these subdomains. This leads to the production of phosphatidylinositol 3-phosphate (PI3P) and to recruitment of PIP3-binding proteins DFCP1 (double FYVE domain containing protein), and WIPI (WD40 repeat protein interacting with phosphoinositides) family proteins. This directs localisation of ATG12-ATG5-ATG16L complex to the nucleation site to perform phagophore elongation (Fig. 1).

The origin of membranes for autophagosome formation has been extensively investigated, and many organelles have been proposed as membrane sources, as well as *de novo* assembly. Endoplasmic reticulum (ER), Golgi, lysosomes, mitochondria, and plasma membrane were all proposed as sources [23,24]. Recent studies suggest that ER contact sites with other organelles become the sites for autophagosome biogenesis [25]. Among them, the Mitochondrial Associated Membrane (MAM), a distinct domain of ER–mitochondria contact sites, was proposed as a place for autophagosome biogenesis [26].

The elongation of phagophores requires lipidation of ATG8 family proteins, including microtubule-associated protein light chain 3 (LC3), to produce LC3II. Before lipidation can take place, LC3 has to be cleaved by cysteine protease ATG4 at its C-terminus, exposing a glycine residue (LC3I form). This is required for LC3-I conjugation to phosphatidylethanoloamine (PE). Lipidated LC3-II (and its family members LC3A, B, C, and GABARAP) become associated with the phagophore membrane and transmembrane protein ATG9 is recruited to further support elongation. ATG9 localises to trans-Golgi network and endosomes and is understood to supply lipid bilayers for phagophore elongation and closure to create the autophagosome [[27], [28], [29], [30]].

The fusion stage with lysosomes to form autolysosomes requires transportation of autophagosomes, formed anywhere in the cell, along microtubules to perinuclear areas of the cell where lysosomes are present by dynein-dependent retrograde transport. The maturation of autophagosomes to autolysosomes depends on the coordinated action of ATG8 family members, tethering factors, Rab GTPases, and SNARE proteins (Synaptic soluble N-ethylmaleimide-sensitive factor attachment protein *receptor*) to mediate fusion of autophagosomes with endolysosomal vesicles [23,24,31]. After fusion, the lysosomal hydrolases degrade the cargo, and the basic building block molecules are recycled to the cytoplasm for reuse, as well as for lysosomal components [32].

## Autophagy in Neurons

Autophagy is particularly important in neurons as they are long-lived terminally differentiated cells, which cannot dilute the accumulated damaged proteins and organelles by cell division. They operate under high metabolic demand, due to repetitive stimulation to process and transmit information, making them uniquely prone to oxidative stress and organelle damage.

The removal of damaged organelles and misfolded or aggregated proteins is important during both neuronal development and during lifelong maintenance of neuronal health. The autophagy pathway has to be adapted to specific demands in neurons due to their highly specialised structure and function: long axons and branched dendrites protruding from the neuronal body, the primary place where biological processes take place. This brings the problem of trafficking over long distances and compartmentalisation. These problems are solved by a specific spatial organisation of autophagy in neurons with different forms of autophagy taking place in different subcellular compartments [33,34]. Autophagosome formation takes place mainly in distal axons, is constitutive, and autophagosomes are transported to soma by retrograde transport during which they fuse with lysosomes. They are rarely detected in dendrites and soma [35,36]. However, dendritic autophagy modulates neuronal excitation and inhibition [37] and dendritic branching [38].

## Autophagy Plays a Vital Role in the Maintenance of Neuronal Homeostasis and Defects in Autophagy Genes Result in Neurodegeneration in Animal Models

Autophagy is crucial to maintain neuronal homeostasis, and its impairment can lead to neurodegeneration. Ubiquitous deletions of core autophagy genes result in neonatal and embryonic lethality; therefore, nervous system-specific knockout mice were used to study the role of autophagy in neurons. The knockdown of autophagy genes in neurons in animal and cellular models results in protein accumulation and neuronal cell loss. Conditional knockdown of *Atg5* in mouse neural precursors led to the loss of Purkinje and cerebral cortical pyramidal cells and axonal swelling in various parts of the brain [39]. Knockdown of Atg7 in the central nervous system of mice resulted in behavioural changes and impairment of coordination. The animals displayed abnormal limb-clasping reflexes and died by 7 months of age. A massive loss of neurons was observed in the cerebral and cerebellar cortices, and there was a build-up of polyubiquitinated proteins in the form of inclusion bodies. The number and size of the inclusion bodies increased as the animals grew older [40]. Moreover, conditional *Atg7* knockdown in Purkinje cells in mice led to neuronal and axonal dystrophy, degeneration of axon terminals, and cell death [41]. As another example, neuron-specific knockdown of FIP200 in mice caused the loss of Purkinje cells and granular cells, spongiosis in the cerebellar white matter, axonal and dendritic degeneration in Purkinje cells, axonal swelling and accumulation of ubiquitin-positive aggregates [42].

## Role of Autophagy in Neuronal Activity, Plasticity and Memory

In addition to its clearing function in homeostasis maintenance, autophagy recently emerged as a regulator of neuronal activity, plasticity and memory. Synaptic plasticity is achieved by structural and functional modifications of synapses, which involve tight regulation of both protein synthesis and degradation in a spatial and temporal manner [43]. The involvement of the ubiquitin-proteosome system (UPS) in this remodelling is well known, but autophagy has emerged as another important player; synaptic proteins and vesicles, postsynaptic receptors and organelles-like mitochondria are carried by autophagosomes at synapses and are degraded by autophagy [44]. Postsynaptic receptors GABAA and AMPA were reported to be degraded by autophagy [37,45] suggesting that autophagy may regulate the balance of neuronal excitation and inhibition, essential for synaptic plasticity and brain function [43]. Presynaptic autophagy was shown to modulate synaptic vesicle numbers and neurotransmission [46]. Mitophagy at the synapses may also be important because synaptic activities demand energy and proper calcium buffering capacity [43,47,48]. Postsynaptic scaffolds PSD-95, PICK1, and SHANK3 were also shown to constitute autophagosome cargo [49]. In addition, several synaptic proteins were shown to modulate the rate of autophagosome biogenesis. The presynaptic proteins Endofilin A (Endo A) and Synaptojanin-1 (Synj1) were found to promote synaptic autophagy [50,51], while active zone protein Bassoon inhibits presynaptic machinery by inhibiting ATG5 [52]. Autophagy involvement in the regulation of synaptic plasticity indicates that autophagy is required for memory formation, and studies showed that autophagy could reverse the age-related memory decline [53].

## Role of Autophagy in Neurodevelopment

Autophagy is also an important regulator of neurodevelopment by negatively regulating axon outgrowth and positively regulating synaptic formation [33,34]. It modulates dendritic branching (arborisation) by facilitating organelle and membrane turnover during branch retraction and extension [38] and/or by decreasing levels of Highwire, a ubiquitin ligase complex, which promotes arborisation, and is needed for normal synaptic morphology during development (and axonal regeneration after injury) [54]. It can also regulate dendritic degeneration [55]. During brain development in mouse embryos increased Atg5 expression is observed at the time of cortical neuronal precursors (NPC) differentiation. In mice with Atg5 deficiency in the cortex - decreased differentiation of NPC and increased neuronal proliferation takes place. This results in impairment of the morphology of cortical neurons [56]. The loss of autophagy due to the deletion of the essential autophagy gene Atg7 in proopiomelanocortin neurons perturbs axon growth [57], while autophagy deficiency in microglia leads to impairment of synaptic pruning and causes social and behavioural defects in mice [58,59].

In summary autophagy has a vital role in neurons by maintaining neuronal homeostasis, protecting from neurodegeneration, and regulating neural activity, plasticity, and neurodevelopment.

## Diseases in Upstream Pathways Controlling Autophagy-the mTOR Pathway

Two pathways that critically control autophagy are the mTORC1 pathway that monitors the availability of nutrients to cells; and the AMPK pathway that monitors energy levels (ATP) in cells [6]. These pathways act proximally in the autophagy pathway, at the UNC51-like kinase (ULK) 1/2 complex. This complex is made up of ULK1 (homologue ULK2), ATG13, ATG101, and FAK family kinase-interacting protein (FIP200). The interaction between ULK1 and ATG13 relies on the regulation by mTOR and AMPK, of ATG13 phosphorylation [60]. When nutrients are in plentiful supply, mTOR phosphorylates ATG13. This inactivates the ULK1 complex and reduces autophagic flux. In contrast, under starvation conditions, the ULK1 complex becomes active as ATG13 is dephosphorylated. The activation of the ULK1 complex and its interaction with AMPK is also dependent on the phosphorylation of ULK1, which is mediated by mTOR. Once ULK1 is activated, FIP200 is phosphorylated. The phosphorylation of FIP200 activates the VPS34 complex, the next step in the autophagy pathway.

Mammalian Target of Rapamycin (mTOR) complex 1 (mTORC1) is a serine threonine kinase, ubiquitously expressed, and a master regulator of cell growth, cellular metabolism, and autophagy. mTORC1 facilitates cellular anabolic processes. However, mTORC1 also inhibits catabolism, including the autophagy pathway. Physiological states that induce autophagy, including nutrient deprivation, reduce the activity of mTORC1. A mediator of the effects of mTORC1 is TFEB; this regulates autophagy through transcription, the UKL1/2, and VPS34 complexes (see Fig. 1). The importance of this pathway is that genetic defects in the mTOR pathway impact on autophagy. In addition, many mTORC1 inhibitor compounds, including drugs already licenced for other conditions, act as autophagy inducers, and some are already used clinically. An illustrative example of a genetic defect in the mTOR pathway is tuberous sclerosis.

## Tuberous Sclerosis

Tuberous Sclerosis, also known as Tuberous Sclerosis Complex (TSC) is a rare genetic disorder caused by germline inactivating mutations in either the TSC1 or TSC2 genes. Affected persons manifest with neurological disease (seizures, intellectual impairment, autism), tumours, which can occur in the brain, heart, skin, and kidney, and pulmonary lymphangioleiomyomatosis. TSC is associated with neurodegeneration of selected neurons [61], and so is included in this review. The tumours are mainly noncancerous (benign), the condition is present from birth, and symptoms usually become apparent in childhood. The tumours can cause a range of additional health problems, including epilepsy, learning difficulties, behaviour problems, including autistic spectrum disorder, and skin problems, such as patches of light-coloured or thickened skin, or acne-like spots on the face. Brain lesions include the benign cerebral cortical lesions called ‘tubers’, nodules, tumours, white matter lesions, hydrocephalus, and hemimegalencephaly. There is limited understanding of the cerebellar pathology; however, a mouse model has been described, with a selective Purkinje cell deletion of *Tsc2* [61]. The abnormalities included a gradual enlargement of the size of the Purkinje cells and apoptotic cell death. Subsequent post-mortem analysis of human cerebellum samples from TSC patients showed Purkinje cell loss by 50%. TSC thus causes significant morbidity in children and adults.

TSC is inherited as an autosomal dominant condition. The disease is due to germline mutations in *TSC1* or *TSC2* [62,63]. The product of the *TSC1* gene, is an interacting partner with tuberin (encoded by the *TSC2* gene, forming a heterodimer [64,65]. The TSC complex is a regulator of the rapamycin-sensitive form of the mTOR kinase (mTORC1) [66]. It is likely that dysregulation of mTORC1 is the route by which the pathogenesis in TSC is mediated. In health, the TSC complex inhibits mTORC1 kinase via tuberin’s GTPase activating domain, mediated by the Rab-like protein Rheb [67,68]. However, with a loss of function of the TSC complex, mTORC1 is activated, and stimulates cap-dependent protein synthesis, cell growth, and proliferation, via phosphorylation of key intermediaries. These include S6 kinase, ribosomal protein S6, and 4EBP1.

In terms of autophagy, mTOR also regulates the phosphorylation status of ATG13, part of the UNC51-like kinase (ULK)1/2 complex. The ULK1-ATG13 interaction modulates autophagy through a complex set of conjugation interactions at the initiation stage of the autophagy pathway. In TSC, mTOR phosphorylates ATG13; this inhibits its interaction with ULK1. The result of this is that the complex is inactivated so that further activation of the autophagy pathway is inhibited. In an elegant series of experiments, Parkhito et al. showed that cells lacking TSC2 have low levels of autophagy under basal and cell stress conditions [69]. The survival of Tsc2-deficient tumour cells was dependent on autophagy induction, thus showing the therapeutic potential of autophagy inducers in TSC. Since the first human studies of the mTOR inhibitor Rapamycin to treat astrocytomas in TSC [70], the mTOR inhibitor Everolimus has been licensed by the UK MHRA and US FDA to treat aspects of TSC, including refractory seizures.

## Diseases in Upstream Pathways Controlling Autophagy-the AMPK Pathway

AMPK can sense energy levels inside cells, in the form of adenosine nucleotides. AMPK is activated when there is a reduction in ATP levels in association with rising levels of AMP and ADP [71]. AMPK has an overall effect of promoting catabolism, including autophagy and inhibiting anabolism. This has the effect of generating ATP. In conditions where nutrients are scarce, AMPK acts as a metabolic checkpoint inhibiting cell growth. This is partly through suppression of mTORC1, via direct phosphorylation of TSC2, but also directly through phosphorylation of the mTORC subunit Raptor (regulatory associated protein of mTOR). This inhibits the mTORC1 kinase complex from phosphorylating its target proteins [72]. The effect of this on ULK1 is to remove its inhibition by mTORC1. In addition to its role in inhibiting cell growth and increasing autophagy through suppression of mTORC1, AMPK can increase autophagy through direct phosphorylation of ULK1. Thus AMPK is able to promote autophagy through dual routes of both direct activations of ULK1 and through modifying the mTORC1-mediated inhibition of ULK1^71^. AMPK can also trigger the removal of dysfunctional mitochondria through the promotion of mitophagy, mediated via ULK1, and can also support the generation of new mitochondria. While autophagy is a survival response to starvation conditions, which inhibit mTOR kinase, diseases that reduce the production of ATP via oxidative phosphorylation can impair autophagy [73]. This report showed elegantly that genetic defects in complex 1 of the respiratory chain were able to suppress mTOR inhibitor-mediated autophagy; on the other hand, interventions that increased mitochondrial function were able to promote autophagy. An example of a respiratory complex 1 defect is Leigh encephalopathy.

## Leigh Encephalopathy

Leigh syndrome is a progressive neurodegenerative disease affecting mostly infants. It is characterised by progressive loss of mental and movement abilities (psychomotor regression) and usually results in death within 2–3 years, often due to respiratory failure. The presentation can be with vomiting, diarrhoea, and difficulty swallowing. These problems cause growth failure and failure to thrive and may cause muscle and movement disorders. These may include weak muscle tone (hypotonia), involuntary muscle contractions (dystonia), and balance problems (ataxia). Sensory deficits causing loss of sensation may also be apparent. Other features may include weakness of eye muscles, rapid involuntary eye movements (nystagmus), optic atrophy, central respiratory failure, hypertrophic cardiomyopathy, and lactic acidosis. It is characterised by bilateral lesions in the brainstem and/or basal ganglia, with an average age of death of 6–7 years [74], and associated with defects in mitochondrial energy production through the oxidative phosphorylation pathway. Mutations in genes encoding subunits of complex 1 of the respiratory chain are commonly reported [75]. This results in defective production of ATP through the oxidative phosphorylation pathway. In normal circumstances, AMPK senses defective production of ATP and promotes autophagy in response. This is indeed the case in patients with Leigh syndrome of late-onset and milder disease severity [76]. However, in a genotype-phenotype correlation study, cells from a patient with the most severe, early-onset disease bearing a mutation in the ND5 gene (13513G > A) was unable to increase autophagic flux in response to reduced ATP production. Currently there are no effective therapies for this devastating condition. There are mouse models of Leigh syndrome, deficient in the mitochondrial respiratory chain subunit Ndufs2 (NADH dehydrogenase ubiquinone) [2]. Critically, rapamycin, a specific mTOR inhibitor, enhanced the survival and attenuated disease progression, including delaying the onset of neurological symptoms. This was thought to be through a metabolic shift towards amino acid catabolism and away from glycolysis, alleviating the build-up of glycolytic intermediates. This does offer the hope that therapies to increase catabolism, including autophagic flux, may be a treatment option in diseases, such as Leigh syndrome, where there is an ATP supply defect, and genotype-phenotype correlation studies support this approach.

## Diseases Affecting Phagophore Nucleation: the PI3KC3 Complex 1

The VPS34 complex is recruited to the autophagosome formation site by the activated ULK1 complex through the phosphorylation of FIP200. The VPS34 complex is made up of the following components: phosphatidylinositol 3-kinase class 3 (VPS34 kinase), Beclin-1, and phosphatidylinositol 3-kinase regulatory subunit 4 (VPS15). These components, once in a complex, form an association with ATG14. Toxic protein aggregates can competitively bind to Beclin-1, preventing phagophore initiation and inhibiting autophagic flux. There are a number of different proteins that contain polyglutamine tract (polyQ) domains, which can interact with Beclin-1 [77]. Diseases, such as Huntington’s (huntingtin protein) and spinocerebellar ataxia 3 (ataxin-3 protein), are caused in part by expanded polyQ tracts, that compete for interaction with Beclin-1 in a length-dependent fashion: the longer the polyQ tract, the more impairment of autophagy initiation. One example of a disease for which pathogenesis is mediated in part by inhibition of phagophore initiation is spinocerebellar ataxia type 3.

## Spinocerebellar Ataxia Type 3 (SCA3)

Also termed Machado-Joseph disease, this disease is characterised by progressive movement disorders, including a gradual loss of balance and falls. There are five types, of which type 1 affects 13% and includes onset in childhood [78]. Its progress can be rapid, with severe rigidity and dystonia. It is caused by mutations in *ATXN3*, encoding Ataxin-3, an enzyme in the ubiquitin-proteasome pathway for removing damaged proteins. The de-ubiquitinating enzyme Ataxin-3 is widely expressed in the brain and can interact with Beclin-1 via its polyglutamine tract (polyQ) domain, protecting it from proteasome-mediated degradation, therefore enabling autophagy. This function for wild type ataxin-3 is abrogated by competing longer polyQ tracts in disease proteins. However, there is another deleterious effect of longer polyQ tracts on ataxin-3 itself, leading to misfolding and clustering together to form aggregates. Neurons appear to be particularly susceptible to these aggregates, leading to cell loss and the characteristic symptoms.

## Diseases Affecting Early Autophagosome Formation

The VPS34 complex is responsible for phosphatidylinositol 3-phosphate (PI3P) being produced. This is done in a part of the ER known as the omegasome [28]. Omegasomes are membrane compartments enriched in PI3P and serve as platforms for the formation of at least some autophagosomes [79]. These have also been termed ‘autophagosome initiation sites’, and they attract proteins that can bind PI3P. PI3P attracts other proteins that mediate its actions. These include WD repeat domain phosphoinositide-interacting proteins (WIPIs) WIPI12 and zinc finger FYVE domain-containing protein 1 (DFCP1). These then migrate to omegasomes. There are diseases that are thought to have their site of pathology at the autophagy initiation step, although the exact mechanisms have not yet been solved. Examples of these are SENDA (static encephalopathy of childhood with neurodegeneration in adulthood), and Mitochondrial membrane protein-associated neurodegeneration (MPAN). Lafora disease is included here as it is correlated with LC3 deficits, which occur before LC3-II formation.

## Mutations in WDR45, Encoding WIPI4 – SENDA (Beta-Propeller Protein-Associated Neurodegeneration (BPAN))

This is a disorder with brain iron accumulation (NBIA) caused by mutations in *WDR45*, encoding a protein called WIPI4 [80,81]. The disease has also been called SENDA (static encephalopathy of childhood with neurodegeneration in adulthood). Children show global developmental delay, then dystonia and dementia in the teens. Cell studies show accumulations of lipidated LC3, and increased levels of LC3 colocalising with mATG9, not usually seen on mature autophagosomes [82]. This suggests that mutations in *WDR45* inhibit the development of the autophagosome, which may be through the regulation of the localisation of mATG9.

## Mitochondrial Membrane Protein-Associated Neurodegeneration (MPAN)

In 2009, a family from Mali was identified with two sisters affected by spastic paraplegia [83b], [83a]. These patients had severe weakness of lower limbs but also muscle atrophy of their upper limbs. Homozygosity mapping localised the candidate gene region to chromosome 19p 13.11-q12, defining this as a distinct form of hereditary spastic paraplegia with amyotrophy, designated SPG43. Four years later, exome sequencing revealed a homozygous missense variant in (c.187G > C; p.Ala63Pro) in *C19orf12*, a gene implicated in neurodegeneration with brain iron accumulation (NBIA) [84], and identified as the cause of a distinct clinical subtype of neurodegeneration with brain iron accumulation two years previously [85]. NBIAs are a mixed set of diseases that present with a gradual development of extrapyramidal symptoms, together with basal ganglia deposition of iron. There are about nine genes that are known to result in NBIA [86], each having similar symptoms. The common clinical features of NBIA are gradual development of dystonia, dysarthria, spasticity, and parkinsonism. Other features may include optic atrophy leading to blindness, retinal degeneration, and peripheral neuropathy. Mitochondrial membrane protein-associated neurodegeneration (MPAN) is the third commonest NBIA and is caused by mutations in the gene *C19orf12* [85]. This disease has its onset in childhood, and additional features may include a motor neuropathy marked by a raised creatine kinase.

The function of the protein has not yet been fully elucidated but has been proposed to have a role in the regulation of autophagy at the sites of autophagosome formation [87]. Overexpression of EGFP-tagged wild type C19orf12 induced the conversion of the autophagic marker LC3 heavy form (LC3I) to the light form (LC3II) compared to cells transfected with EGFP empty vector, and reduction of p62, indicating the elevation of basal autophagy levels. In contrast, the overexpression of the vectors carrying mutant forms of the protein was unable to gain their correct internal localisation and failed to promote autophagy induction. As a result, basal autophagy activity was stable despite treatment with oxidative stressors.

## Lafora Disease

Lafora Disease (LD) is a rare, autosomal recessive, neurodegenerative disorder characterised by myoclonic epilepsy, presenting between 8 and 18 years with tonic-clonic seizures, myoclonus, drop attacks and visual hallucinations, rapidly leading to cognitive decline with dementia, apraxia, aphasia, and vision loss [88]. Most patients have mutations in one of two genes, which are *EPM2A*, encoding a protein phosphatase called Laforin, and *EPM2B* (Malin), an E3 ubiquitin ligase [89]. In Lafora disease, there are Lafora bodies (inclusion bodies) that are found in the cytosol of neurons, and also in the cytoplasm of cells from tissues with high carbohydrate metabolism (liver, heart, skeletal muscle). These inclusion bodies are made up of polyglucosans, which are an abnormal, heavily phosphorylated, and poorly branched form of glycogen.

Laforin interacts with malin to form a complex; this initiates ubiquitination and degradation of proteins that function to regulate the synthesis of glycogen. Laforin can also act as a glycogen phosphatase, preventing glycogen from being phosphorylated. This has the effect of stopping too much glycogen from being phosphorylated. This is useful as it inhibits the formation of glycogen polymers that are prone to aggregation to form Lafora bodies. In the related malin knockout mouse model, glycogen accumulation was demonstrated and accounted for the neurodegeneration and functional consequences [90]. It should also be noted, however, that defects in autophagosome formation have also been demonstrated [90]. Human fibroblasts from LD patients lacking laforin were found to have reduced levels of LC3-II compared with healthy fibroblasts. This was confirmed with mouse embryonic fibroblast cells from laforin null mice and suggested that the insufficiency of laforin-malin complexes will inhibit the development of the autophagosome [91]. Treatments have traditionally centred on antiepileptic therapies. However, efforts are now turning to the downregulation of brain glycogen synthesis and disease gene replacement [92].

## Phagophore Membrane Elongation

Once the VPS34 complex has generated PI3P, PI3P binding proteins, such as WIPI family proteins are recruited [58]. Their target is a membrane resident phospholipid (phosphatidylethanoloamine (PE)). The conversion of soluble LC3-I to lipid-bound LC3-II is an important step in the formation of autophagosomes [28]. Single-gene defects that affect the autophagy pathway at this stage include some of the hereditary childhood ataxias, such as that due to mutations in the ATG5 gene, as ATG5 deficiency can result in a defect in LC3 lipidation.

## Hereditary Childhood Ataxia due to Mutations in ATG5

Ataxia is a rare disorder affecting balance and coordination, so that affected patients find walking difficult and suffer falls. This disease is associated with abnormalities in the cerebellum and specifically Purkinje cells. Children with ataxia may have associated coordination problems and learning difficulties. There are over 60 genes that are known to cause ataxia. Two Turkish siblings who presented with ataxia and developmental delay were found to have a homozygous mutation in the gene encoding ATG5 [93]. The reported E122D mutation in ATG5 is thought to involve the ATG12-ATG5 interaction site. Disease affected patient cells showed significantly reduced ATG12-ATG5 conjugate, raising the possibility that autophagy may be impaired via inhibition of this conjugation. Perhaps not surprisingly, these same mutant cells showed reduced autophagic flux.

## Hereditary Spastic Paraplegia (SPG 49) due to TECPR2 Mutations

Hereditary spastic paraplegias (HSPs) present with increased tone and reduced muscle strength in the lower limbs; and sometimes other complications, such as ataxia, dementia, or amyotrophy. There appears to be a degeneration of motor neurons occurring in a retrograde fashion. There are more than 70 genes known to cause this; one of these is mutations in the gene encoding tectonin beta-propeller repeat-containing protein 2 (*TECPR2*) [94]. Affected patients have been reported with learning difficulties, central hypoventilation, and gastro-oesophageal reflux. TECPR2 is known to interact with ATG8 and appears to work as a scaffold for the COPII coat protein SEC24D [95]. The export of cargo proteins from the endoplasmic reticulum (ER) is mediated by COPII-coated vesicles that form at endoplasmic reticulum exit sites (ERES). The COPII components have isoforms: these may deliver selection to the export of cargoes, particularly in relation to SEC24. TECPR2 is thought to be necessary for the proper working of ER exit sites and export from the ER. This occurs with the assistance of lipidated LC3C.

The ATG8 family of proteins is able to recruit autophagy proteins containing domains that interact with LC3. They further have a function in the extension and closing of the phagophore membrane. LC3 is also needed to bring labelled cargo into autophagosomes via LC3 interaction region cargo receptors. The autophagosomal membrane elongates by appropriating other cellular membranes, such as the plasma membrane, mitochondrial, recycling endosomes, and the Golgi complex [28]. Vesicles that contain ATG proteins are involved in delivering some of the membranes. The membrane of the autophagosome finally becomes continuous, creating a bi-layered vesicle. The next step is the removal of the ATG proteins, which matures (including stripping of the ATG proteins), before fusing with a lysosome. During this process, coat proteins, such as Adaptor protein-4, are recruited to membranes with their cargo. ATG9 is a specific cargo of adaptor protein-4 [96]. Adaptor protein-4 promotes signal-mediated export of ATG9A from the trans-Golgi network to the peripheral cytoplasm, contributing to lipidation of LC3B and maturation of preautophagosomal structures.

## Hereditary Spastic Paraplegia due to Adaptor Protein-4 Deficiency

This disease presents with many of the features of hereditary spastic paraplegia, as already outlined previously. It is caused by mutations in genes that encode subunits of adaptor-protein 4 (SPG47, SPG52) [97]. Additional features of patients include early floppiness that graduates to stiffness, learning difficulties, and reduced brain volume, thin corpus callosum, and seizures. The pathology is poorly understood, but mouse models show many of the neurological features of patients and show ATG9 mislocalisation; ATG9 shows expression throughout the *trans-*Golgi network and reduced expression in peripheral cytoplasm. There appears to be an accumulation of toxic proteins in neurons. Impaired autophagic degradation of protein aggregates is likely to contribute to the neuroaxonal dystrophy in this disease.

## Diseases Affecting Cargo Recognition or Delivery for Degradation

Selective autophagy is achieved by labelling of cargo by ubiquitin chains, recognised by autophagy receptors. These connect autophagic cargo to the autophagosome membrane through their LC3-interactions. ULK1 is activated independently of mTOR, and there is some evidence that huntingtin, the wild type protein product of the Huntingtin gene, may be the molecular link [98]. Huntingtin may also mediate the interaction of p62 and LC3 with ubiquitin polypeptides. This links the recognition of cargo with selective autophagy [28].

## p62 Mutations

It has been estimated that 30% of newly made proteins are misfolded in health [1]. The ubiquitin-proteosome system (UPS) and the autophagy pathway are recognised to maintain homeostasis in cells. Although distinct, the proteolytic pathways interact and are important for the survival of cells both in health and stress [99]. There are shared proteins, including HttQ74 and SQSTM1/p62. This is a classical receptor of autophagy and important for ubiquitinated protein degradation. SQSTM1/p62 is a scaffold protein, and functions to anchor the ubiquitinated proteins to the autophagosome membrane, promoting degradation of unwanted molecules [100].

Mutations in *SQSTM1/p62* are found in frontotemporal dementia (FTD) and amyotrophic lateral sclerosis in adults (ALS) [101]. However, recently, biallelic loss-of-function variants were identified in *SQSTM1/p62* in nine affected individuals from four families with childhood- or adolescence-onset neurodegenerative disorder characterised by gait abnormalities, ataxia, dysarthria, dystonia, vertical gaze palsy, and cognitive decline [102]. They reported evidence of a defect in the early response to mitochondrial depolarisation and autophagosome formation. In a study of fibroblasts from FTD patients carrying two independent pathogenic mutations in the p62 gene, p62 deficiency was found to be associated with inhibited complex I mitochondrial respiration due to lack of NADH for the electron transport chain [103]. These findings highlight the implication of energy metabolism in pathophysiological events associated with p62 deficiency.

## Huntington Disease

Huntington disease (HD) is a progressive, neurodegenerative disorder that causes uncontrolled movements, emotional problems, and loss of thinking ability (cognition). It is estimated to affect between 3 and 7 per 100,000 people of European ancestry and is less common in people of Japanese, Chinese, and African descent. The most common form is an adult onset, but there is a juvenile form that begins in childhood or adolescence. In addition to the features listed above, it also causes slow movements, clumsiness, frequent falls, rigidity, slurred speech, and drooling. School performance declines as thinking and reasoning abilities become impaired. Seizures occur in 30–50% of children with this condition. Disease progression is usually faster in childhood-onset compared with adult-onset disease.

This is an autosomal dominant trinucleotide repeat disorder, caused by N-terminal polyglutamine tract expansions (blocks of cytosine-adenine-guanine trinucleotides in various multiples of repeats). The trinucleotide usually may show multiples up to 10–35 times. However, patients with Huntington disease may show many more repeats, and there appears to be an inverse relationship between the number of tandem repeats and the age of onset; the childhood-onset forms of the disease have the highest number of repeats. There is some evidence that the predominant action of the HD mutation is a toxic gain of function [104]. Until recently, there has been no disease-modifying therapy available for HD.

Various steps in the autophagy pathway are altered in HD [105]. These include a defect in cargo loading, trafficking of autophagosomes, and decreased fusion between autophagosomes and lysosomes, leading to a build-up of toxic materials in the cytoplasm and empty autophagosomes. The primary defect may be in the ability of autophagic vacuoles to recognise cytosolic cargo in HD cells [106]. Autophagosomes fail to efficiently trap cytosolic cargo in their lumen. This failure to properly engulf polyglutamine tract aggregates, or inefficient cargo loading, leads to slower turnover, decay, and accumulation inside HD cells.

Wild-type huntingtin regulates autophagy by binding and releasing ULK1 from mTOR and supporting the p62 adaptor interaction with LC3^108^. Mutated huntingtin, on the other hand, forms aggregates and is likely toxic, but can be cleared by autophagy. In HD cells containing polyglutamine aggregates, these aggregates sequester mTOR, resulting in decreased mTOR activity and an increase in autophagy, leading to more rapid clearance of the soluble protein in such cells [104]. In contrast, cells expressing nonaggregated mutant polyglutamine proteins seem to correlate with greater toxicity and autophagy inhibition. The soluble mutant huntingtin protein competes with the deubiquitinating enzyme Ataxin-3, which is protective, for Beclin-1 interaction. This competitive binding inhibits autophagy, and reduced expression of LC3 and Beclin-1 have been found in protein extracts from brain samples of patients with HD [107].

Rapamycin, an autophagy inducer, has been shown to enhance mutant huntingtin fragment clearance and attenuate toxicity, and in fact, may have wider applications for enhancing the autophagic clearance of a variety of proteins and reduces their toxicity [108]. A recent early-phase trial of an antisense oligonucleotide designed to inhibit *HTT* mRNA has demonstrated reductions of mutant huntingtin in human patients and holds promise for future treatments for this devastating disease [109].

## Autophagosome Maturation and Fusion with Lysosomes

Once the autophagosomal membrane has formed a bi-layered vesicle (autophagosome), the ATG9 proteins are stripped out before fusion with the lysosome. Before that can occur, machinery has to be recruited that will approximate the lysosomes (microtubule-based kinesin motors), and that will fuse the autophagosome with the lysosome [28]. This machinery includes SNAREs (syntaxin 17 and synaptosomal-associated protein 29 (SNAP29) on the autophagosome, vesicle-associated membrane protein 8 (VAMP8) on the lysosome, and the homotypic protein fusion and sorting (HOPS) complex, which mediates membrane tethering to support SNARE-mediated fusion. Disorders of the pathway thought to be at this stage include a rare form of leukoencephalopathy, and VICI syndrome.

## Leukoencephalopathies

Leukoencephalopathy refers to diseases affecting the white matter of the brain. White matter consists mainly of myelinated axons that carry the nerve impulses between the neuronal cell bodies in the gray matter. White matter disease affects the networking between neuronal cell body centres in the gray matter. Myelin is disrupted, either through destruction or from biochemically abnormal myelin production. White matter disorders tend to present in childhood rather than infancy, with initially normal cognitive function. Demyelination may be early, causing peripheral neuropathy, and spasticity may be severe. Seizures tend to be a later feature. White matter diseases may present with motor signs, such as gait disturbance, ataxia, and spasticity, and with optic atrophy and blindness. Among many causes, rare monogenic defects in the autophagy pathway include mutations in vacuolar sorting protein 11 (VPS11), needed for autophagosome membrane fusion.

VPS11 protein, encoded by the *VPS11* gene, is a core component of homolytic fusion and protein sorting (HOPS) and class C core vacuole/endosome tethering (CORVET) protein complexes, involved in membrane trafficking and fusion of the lysosomes and endosomes [110]. The C846G *SNF8* mutation causes aberrant ubiquitination and accelerated turnover of VPS11 protein, as well as compromised VPS11-VPS18 complex assembly. Reduced VPS11 expression causes impaired autophagic flux, which impairs autophagy in human cells.

## VICI Syndrome

Mutations *EPG5* cause VICI syndrome, an infancy onset disorder [6]. The key clinical features include underdevelopment of the corpus callosum, progressively small head, cataracts, cardiomyopathy, immunodeficiencies, hypopigmentation, and global delay. Other important features include floppiness due to muscle disease. There is evidence of Purkinje cell loss and diffuse cerebral atrophy. This, together with loss of previously acquired skills, points to neurodegeneration. Children succumb to heart failure and infections. A large case series of 50 children from 30 families showed that neurodegeneration is a primary effect of EPG5 mutation, not secondary to other neurodevelopmental defects [111].

*EPG5* is the human homologue of the metazoan-specific autophagy gene *epg-*5, encoding a key autophagy regulator (ectopic P-granules autophagy protein 5), implicated in the formation of autolysosomes [112]. Mutant Epg5 in knockdown models resulted in a build-up of abnormal autolysosomes, suggesting that Epg5 is also involved in the later stages of autophagy, including autophagosome-lysosome fusion and proteolysis [113,114]. Investigation of muscle and skin samples from affected patients showed a build-up of LC3-positive autophagy vesicles and linking proteins NBR1 and p62. This suggests defective autophagic flux at the late stages of autophagy. The clearance of intracellular pathogens is dependent on an intact autophagy pathway [115], and the failure to do this is likely to impair immunity and explain the recurrent infections seen in patients.

## Hereditary Spastic Paraplegia due to CCT5 Mutations

This is an autosomal recessive HSP, presenting with a peripheral sensory deficit with generalised weakness. The neuropathology involves posterior column atrophy, and the disease is due to mutations in CCT5 [116]. This disease was reported in a Moroccan family where there were four siblings from age 1–5 years, with a severe mutilating sensory neuropathy with sensory paraplegia. These children had spasticity of their legs, exaggerated reflexes, loss of sensation in the distal parts of both upper and lower limbs, and deep perforating ulcers of hands and feet. Unfortunately, the sensory neuropathy was rapidly progressive. Nerve conduction studies confirmed a sensory axonal neuropathy. This gene encodes the epsilon subunit of the cytosolic chaperonin-containing t-complex 1. The complex consists of two identical stacked rings; unfolded peptide chains enter the central cavity of the complex and are folded in an ATP-dependent manner. The complex folds cytoskeletal proteins, such as actin and tubulin, but also proteins required for autophagy. Mutations in the gene encoding the CCT5 subunit, lead to the accumulation and aggregation of misfolded proteins required for autophagy machinery [117]. CCT5 also supports the degradation of autophagosomes by modulating lysosomal function.

## Ataxia due to SNX14 Mutations

Twelve families were described with ataxia, coarse facial features, and intellectual disability, and in addition, had cerebellar atrophy [118]. These families were found to have truncating mutations of sorting nexin 14 (*SNX14*), encoding a ubiquitously expressed modular PX-domain-containing sorting factor. SNX14 interacts with phosphatidylinositol 3,5-bisphosphate (PtdIns [3,5]P2) localising to late endosomes/lysosomes, which contain PtdIns [3,5]P2. Neural progenitor cells with SNX14 mutations showed engorged lysosomes and a slower autophagosomal clearance rate on the induction of starvation. The data suggest that without SNX14, there is a disruption of autophagosome clearance through impairment of the function of lysosomes.

## Charcot Marie Tooth Disease due to Mutations in Dynamins

Charcot Marie Tooth (CMT), also known as Hereditary Sensory and Motor Neuropathy (HSMN), or peroneal muscular atrophy, is the name given to a group of inherited conditions that damage the peripheral nerves. Symptoms usually appear between 5 and 15 years of age, although they may be delayed until adulthood. They can present with muscle weakness in the feet, ankles, legs and hands, abnormal gait, either high arched or very flat feet, and numbness in the feet, arms, and hands. This is a progressive condition with nerve loss, and affects about 1 in 2500 people.

CMT is caused by mutations in genes that produce proteins involved in the structure and function of either the peripheral nerve axon or the myelin sheath. All the mutations affect the normal functioning of peripheral nerves, causing them to degenerate. Motor nerve degeneration causes muscle weakness and atrophy in the extremities, and sometimes this is accompanied by sensory nerve degeneration, which causes reduced ability to feel heat, cold, and pain.

Over 70 genes are known to cause this disease, but at least 2 genes encode proteins involved in the autophagy pathway. Mutations in dynamin 2 also cause CMT associated with neutropenia and early-onset cataracts, together with facial weakness and ptosis. Dynamin is a 100 kDa GTPase involved in membrane and cytoskeleton remodelling. In the central nervous system, dynamin-2 has a role in endocytosis at the post-synaptic membrane, in neurosecretion, and in neural process extension. Most of the dynamin-2 CMT-related mutations are located in the N-terminal region of the pleckstrin homology domain in a region that is involved in the insertion of dynamin into lipid membranes, and some have been shown to be unable to bind phospholipids. The protein also appears to be important for lysosomal/autolysosomal acidification. Loss of function mutations is associated with the accumulation of immature autophagosomes [119].

## Spinal Muscular Atrophy due to Mutations in DYNC1H1

Spinal muscular atrophy (SMA) is a genetic disease with spinal cord motor neuronal degeneration, leading to severe muscle weakness, particularly of the lower limbs [120]. Affected children had a waddling, wide-based gait from infancy, leading to difficulty in running. The upper limbs were unaffected, and the weakness in the lower limbs did not seem to be progressive. The cause was identified as mutations in Dynein cytoplasmic 1 heavy chain 1 *(DYNC1H1)*. This gene encodes a critical subunit of the cytoplasmic dynein complex. Dyneins are a family of cytoskeletal motor proteins (ATPases) that transport cellular cargos along with microtubular networks. They are essential for intracellular motility, such as retrograde axonal transport, protein sorting, and redistribution of organelles, such as lysosomes. A key role is in the autophagosome migration phase of autophagosome-lysosome fusion: autophagosomes migrate to the locations of lysosomes via the microtubular network. Disruption of the dynein protein likely reduces the intracellular transport of autophagosomes, and also of other proteins, allowing them to accumulate and form aggregates.

## Hereditary Spastic Paraplegia due to Mutations in SPG11 and SPG15

As discussed above, hereditary spastic paraplegias (HSPs) are a heterogeneous group of inherited neurodegenerative and neurodevelopmental disorders characterised by retrograde neurodegeneration of the longest corticospinal motor neurons. The commonest clinical presentation is with spastic paraparesis and urinary incontinence, which manifest as lower-limb hypertonia, weakness, and reduced vibration sense. There are at least 70 different genetic loci identified (SPF 1–72) [121]. However, the commonest genetic cause (70%) is due to mutations in the genes encoding the proteins SPG11 (*SPG11)* and SPG15 (*ZFYVE26*). These cause progressive stiffness of the legs, developmental delay, pigmentary retinopathy, and amyotrophy. *SPG11* and *SPG15* encode spatacsin and spastizin, respectively, which are needed for lysosomal development. There is an interaction with the AP5 complex, which is needed for late endosome membrane sorting [122]. The AP5 complex is a member of a family of heterotetrametic adaptor proteins that associates with SPG11 and SPG15 to form a coat-like complex, with AP5 involved in protein sorting, SPG15 facilitating the docking of the coat onto membranes by interacting with PI3P, and SPG11 (possibly with SPG15) forming a scaffold. The SPG protein spastizin is encoded by the gene *ZFYVE26* and colocalises with early endosomes, the endoplasmic reticulum, microtubules, and vesicles involved in protein trafficking. Spastizin interacts with the autophagy-related Beclin 1-UVRAG-Rubicon complex, a protein required for cytokinesis and autophagy [123]. In cells lacking spastizin, there is an impairment of autophagosome maturation and an accumulation of immature autophagosomes. These defects were also observed in neuronal cells and may be responsible for the thinning of the corpus callosum seen in many patients.

## Current Challenges

One of the great challenges of the field is to translate the rapid advances in our understanding of autophagy, into treatments for children affected by single-gene disorders. Dysfunctional autophagy has been reported in almost all the neurodegenerative diseases studied, including common adult disorders, such as Alzheimer’s and Parkinson’s [30,[124], [125], [126]]. Common therapeutic strategies include the induction of autophagy to restore proteostasis, regardless of the nature of the different misfolded proteins or aggregates in these disorders. Driving autophagic flux is neuroprotective in several transgenic mouse models of neurodegenerative diseases and other neurological disorders [30,124,125,127]. For example, genetic and pharmacological enhancement of autophagy was able to reduce the number of synuclein aggregates, oxidative damage, and improve neuronal viability [124,125,128]. Likewise, pharmacological inducers of autophagy could enhance the clearance of mutant huntingtin and ameliorate the disease phenotypes in mouse models of HD [30,124,125,127,129,130].

In spite of the robust literature on the role of autophagy in neurodegenerative disease, a lack of clinically relevant data for autophagy inducers has hampered clinical trial efforts. Moreover, emerging studies indicate the autophagy modulators can have cell-type-specific action and efficacy [131], and there is no clear information about the nature of the candidate autophagy-inducing drug for clinical translation. It is highly unlikely that pharmaceutical companies will invest in separate clinical trials for the 150 or so rare childhood-onset neurodegenerative disorders. There is, therefore, a need to develop potent autophagy inducers as a shared mechanism in order to restore functional autophagy and improve neuronal viability across several rare neurodegenerative diseases.

There are over 20 FDA-approved drugs known to induce autophagy, and that cross the blood-brain barrier. These are drugs that are licensed for use in other conditions, but that also have known autophagy-inducing properties. There are no obvious side-effects that are known from the therapeutic stimulation of autophagy in neurodegenerative diseases where activation of autophagy acts as a protective pathway. One theoretical concern is that stimulation of autophagy could decrease the levels of mitochondria (via mitophagy), and affect respiration. However, this scenario has never been seen in transgenic mice or patients because the mitochondrial load has to be significantly lowered by 75% in order to have an impact on respiration [104,127]. Any focus on repurposed drugs will probably have to investigate a mTORC1-independent mechanism rather than those agents that inhibit mTORC1, such as rapamycin. This aspect is particularly important and clinically relevant for long-term drug administration in neurodegenerative disease patients because mTORC1 governs critical cellular functions, such as cell growth and translation. The mTORC inhibitors, therefore, may not be practical solutions for inducing autophagy long-term.

For diseases where there is an accumulation of toxic aggregates due to the gain of function mechanisms, another approach is to target the causative gene with antisense therapy. The current example of this approach is in Huntington disease, where a CAG nucleotide repeat expansion in the huntingtin gene results in the production and accumulation of mutant protein. Recently, the results of treatment with an antisense oligonucleotide have been published, designed to reduce concentrations of huntingtin mRNA [109]. Intrathecal administration to patients with Huntington’s disease was shown to be safe, with dose-dependent reductions in mutant huntingtin. This is a really exciting approach that is likely to be generalisable to other autophagy defects caused by gain of function mechanisms.

Finally, gene replacement therapy is gaining interest as an option for single-gene disorders, causing a deficiency in an essential protein for the autophagy pathway. Gene therapy that delivers a working version of a gene would be an attractive strategic approach to some of these diseases, such as Lafora disease [92]. The most popular strategy that has been developed to introduce a transgene is the use of the viral vector adeno-associated virus. This is the vector of choice as it is nonpathogenic, is long-lasting so that only one treatment might be needed, and crosses the blood-brain barrier, making intravenous delivery possible, although intrathecal delivery is likely to be a preferred route. Any gene therapy to arrest neurodegeneration would need to be delivered before extensive central nervous system damage has occurred.
